# Differential predictive value of phase angle for malnutrition and physical impairment in respiratory patients: the moderating role of sarcopenia

**DOI:** 10.3389/fnut.2026.1828775

**Published:** 2026-07-03

**Authors:** Cai Li, Juan He, Li Ma, Yijing Li, Jingyuan Wang, Xiaohong Lu, Hongxia Sun, Miao Tuo, Lili Wei

**Affiliations:** 1 Department of Clinical Nutrition, The Affiliated Hospital of Qingdao University, Qingdao, China; 2 Department of Nursing, The Affiliated Hospital of Qingdao University, Qingdao, China; 3 Department of Cardiology, The Affiliated Hospital of Qingdao University, Qingdao, China; 4 Department of Respiratory Medicine, The Affiliated Hospital of Qingdao University, Qingdao, China; 5 Office of the Dean, The Affiliated Hospital of Qingdao University, Qingdao, China

**Keywords:** AWGS 2025, GLIM, malnutrition, phase angle, physical performance, respiratory disease, sarcopenia

## Abstract

**Background:**

Phase angle (PhA) is a vital indicator of nutritional and physical performance, yet its utility may vary across different clinical phenotypes. This study aimed to determine the associations between PhA, malnutrition, and physical performance, and to evaluate whether these relationships are contingent upon sarcopenia status.

**Methods:**

This study enrolled patients from the Department of Respiratory Medicine from March to September 2025. PhA was assessed using bioelectrical impedance analysis. Malnutrition and sarcopenia were diagnosed based on the Global Leadership Initiative on Malnutrition criteria and the Asian Working Group for Sarcopenia 2025, respectively. Physical performance was evaluated using the Short Physical Performance Battery score. The association between PhA and clinical outcomes, and potential moderating effect of sarcopenia, were analyzed using multivariable regression and interaction analysis.

**Results:**

A total of 235 patients were analyzed (mean PhA: 4.51 ± 0.9°), including 114 with sarcopenia. Prevalence of malnutrition and impaired physical performance was 43.4 and 40.9%, respectively. After adjusting for potential confounders, interaction analysis revealed that the association between PhA and malnutrition was significantly modulated by the presence of sarcopenia (*P _interaction_* = 0.035). Subgroup analysis revealed that a significant decrease in PhA was associated with an increased risk of malnutrition in the sarcopenia group (OR = 3.11, 95% CI: 1.52–6.33, *p* < 0.001), whereas no such association was observed in the non-sarcopenia group (OR = 1.35, 95% CI: 0.52–3.52, *p* = 0.52). Conversely, each 1°PhA decrease was associated with an increased risk of impaired physical function (OR = 4.59, 95% CI: 2.44–8.62, *p* < 0.001), regardless of sarcopenia status (*P _interaction_* = 0.561).

**Conclusion:**

The association between PhA and malnutrition is primarily driven and amplified by sarcopenia. However, PhA remains a robust, independent predictor of impaired physical performance across all respiratory patients regardless of sarcopenia status.

## 1 Introduction

Malnutrition and impaired physical performance are frequent complications in chronic respiratory diseases including Chronic Obstructive Pulmonary Disease (COPD), interstitial lung disease (ILD) and various infectious lung diseases. These comorbidities not only predict a poorer prognosis but also exacerbate the severity of the underlying diseases. According to the Global Leadership Initiative on Malnutrition (GLIM) criteria, approximately 22.6% of patients with COPD are malnourished (1). Similarly, impaired physical performance is a primary concern in patients with COPD, ILD, and asthma, and is strongly linked to adverse clinical outcomes (2, 3). As physical performance declines, patients often restrict their daily activities, leading to a vicious cycle of functional deconditioning and disability. To quantify this impairment, the Short Physical Performance Battery (SPPB), comprising gait speed, a balance test, and a chair-stand test, is frequently employed. Furthermore, incorporating the SPPB into the Body-mass index, airflow Obstruction, Dyspnea, and Exercise capacity index maintains its predictive accuracy for all-cause mortality, suggesting that its adoption in clinical practice could enhance the comprehensive risk assessment and prognostic stratification in respiratory diseases (4). Sarcopenia is a common comorbidity in these patients. A meta-analysis indicates that the prevalence of sarcopenia ranges from 15.5 to 34%, which correlates with the severity of respiratory impairment (5). Sarcopenia significantly overlaps with both malnutrition and physical performance decline, forming a complex relationship. According to the GLIM criteria, reduced muscle mass is a phenotypic hallmark of malnutrition; simultaneously, chronic malnutrition is a primary driver in the pathogenesis of sarcopenia. Meanwhile malnutrition is also a cause of sarcopenia. Concurrently, the Asian Working Group for Sarcopenia 2025 (AWGS 2025) identifies poor physical performance as a key outcome of sarcopenia (6). Malnutrition, sarcopenia, and impaired physical performance often coexist and exacerbate one another, creating a deleterious cycle that complicated the clinical course of chronic respiratory patients. Due to their overlapping diagnostic features and shared pathophysiological underpinnings, identifying a reliable biological indicator is essential.

Bioelectrical impedance analysis (BIA) is a non-invasive and efficient technique that provides electrical impedance data, specifically resistance (R) and reactance (Xc). From these, the phase angle (PhA) is calculated by the equation: PhA = arctan (Xc/R) × (180/*π*) (7). As a biomarker of cell membrane integrity and cellular vitality, PhA reflect the balance between intracellular and extracellular water (8). Low PhA levels are associated with frailty, falls, incident disability and mortality (9). In addition to its correlation with body cell mass (BCM) and fat-free mass (FFM), PhA is inversely associated with visceral adipose tissue (VAT) (10). Recent studies have demonstrated that lower PhA is associated with reduced aerobic capacity, impaired gait, and higher biological fitness age (11). However, its predictive value for nutritional status remains inconsistent across diverse clinical populations (12). The loss of muscle mass characteristic of sarcopenia may significantly modulate PhA values, potentially altering its predictive accuracy for malnutrition or physical function. As a biomarker of cell membrane integrity and BCM, PhA values are intrinsically linked to muscle quality and quantity. It is plausible that in patients with sarcopenia, the baseline reduction in PhA driven primarily by chronic muscle depletion may attenuate its sensitivity to acute nutritional shifts. This potential confounding effect could mask or alter the predictive accuracy of PhA for malnutrition in this specific subgroup. Consequently, it remains unclear whether the presence of sarcopenia influences the predictive utility of PhA for these clinical outcomes in respiratory patients.

In this study, we aimed to investigate the correlation between PhA, malnutrition and physical performance in patients with respiratory diseases. Crucially, we assessed whether these associations are modified by the presence of sarcopenia by formally testing for statistical interaction. Furthermore, we sought to determine the optimal PhA cutoff values for the identification of these adverse clinical outcomes.

## 2 Methods

### 2.1 Participants

**Figure 1:**
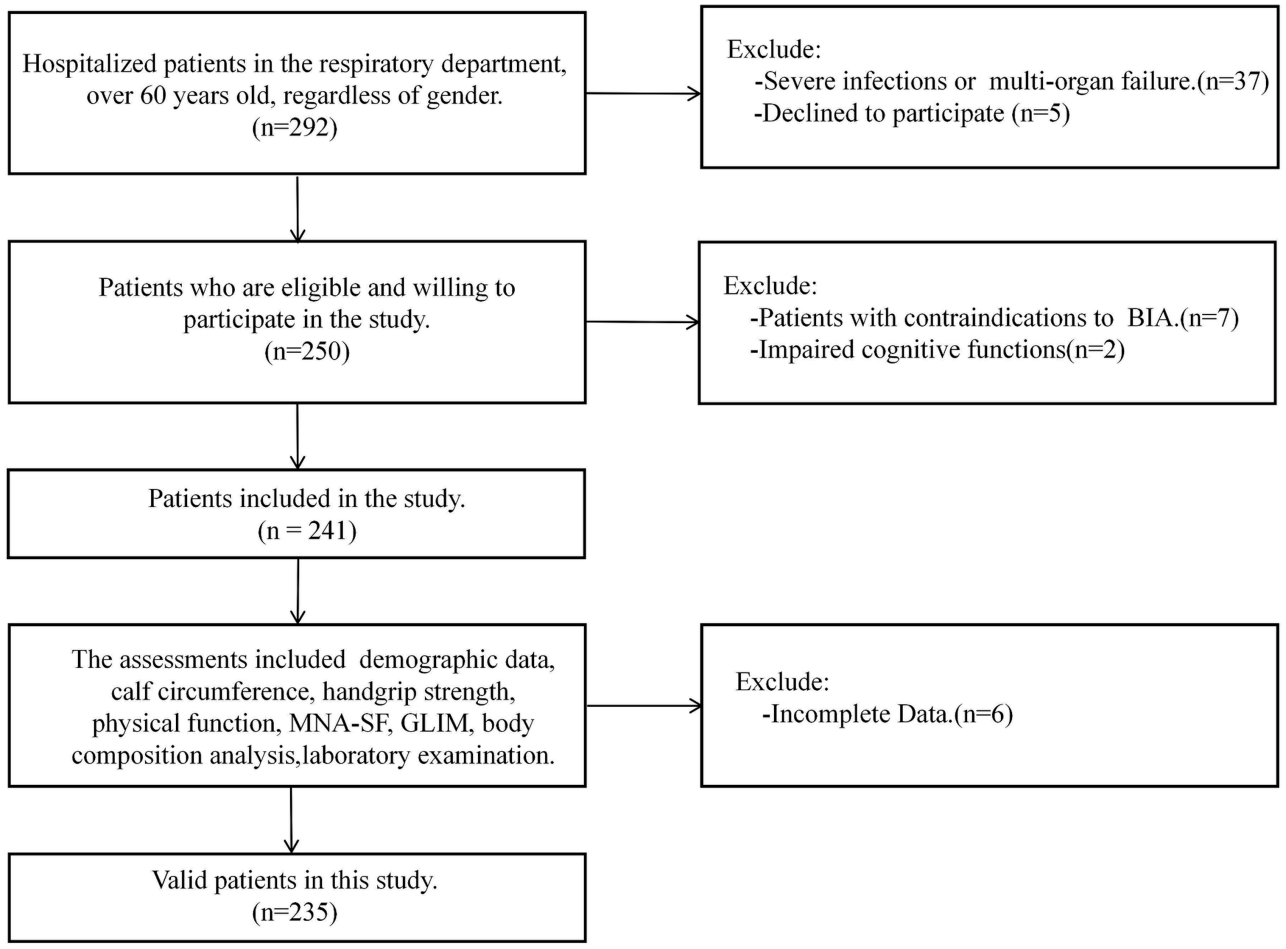
The study’s flow diagram.

A total of 235 patients aged over 60 years were recruited from the Department of Respiratory Medicine, the Affiliated Hospital of Qingdao University between March and September 2025. Among them, 96 (40.9%) were female. Participants were categorized into five groups based on their primary diagnosis: acute exacerbation of chronic obstructive pulmonary disease (AECOPD); interstitial lung disease (ILD); lung space-occupying lesions (LSOL, including nodules and malignancies); Pneumonia, acute infections (e.g., community-acquired pneumonia, lung abscess) excluding cases secondary to COPD, ILD, or lung cancer; and Others, such as bronchiectasis, bronchial asthma, and pulmonary embolism. We determined primary respiratory diagnoses based on the main clinical diagnosis recorded at discharge. Attending physicians established these diagnoses in strict accordance with international standard guidelines. Demographics such as age, gender, height, weight, ethnicity, smoking status, alcohol use, and exercise frequency were collected. The exclusion criteria were as follows: (1) Severe infections or multi-organ failure; (2) Patients with contraindications to BIA, such as patients implanted electronic medical devices, severe edema, and amputation; (3) Impaired cognitive functions; (4) Declined to participate; (5) Incomplete data. This study was approved by the Ethics Committee of the Affiliated Hospital of Qingdao University (approval number: QYFYWZLL30261) and registered at the Chinese Clinical Trial Registry (ChiCTR2500113377). Written informed consent was obtained from all participants prior to enrollment. This study was conducted in accordance with the Declaration of Helsinki and the Ethical Guidelines for Life Sciences and Medical Research Involving Human Subjects. The experimental procedure is illustrated in Figure 1.

### 2.2 Data collection

#### 2.2.1 Body composition

Body composition parameters were assessed using a multi-frequency segmental bioelectrical impedance analyzer (InBody S10, Biospace, Seoul, South Korea). Given that our study population consisted of older Asian adults, we calculated ASM using a previously validated formula: 0.276 × H^2^/R₂₅₀ + 1.151 × sex + 0.059 × Xc_5_ + 0.429 (13). *Where: H is height in cm; R_250_ is the resistance at 250 kHz (Ω); Xc_5_ is the reactance at 5 kHz (Ω); sex: male = 1, female = 0*. ASMI was subsequently calculated as ASM divided by height squared (kg/m^2^). The device measures impedance at six discrete frequencies (1, 5, 50, 250, 500, and 1,000 kHz). Whole-body PhA at 50 kHz was estimated from right-side segmental impedance (right arm, trunk, right leg), as recommended by the manufacturer and based on the assumption of bilateral symmetry. PhA was calculated as follows: arctan (Xc/R) × (180/*π*). Before measurement, patients were required to refrain from strenuous activity for 24 h. Strenuous activity was defined as physical activities with an intensity exceeding 6 metabolic equivalents, such as running, competitive sports, swimming, or heavy weightlifting. Meanwhile, participants were allowed to maintain their unrestricted routine daily activities. BIA assessments were standardized to the late afternoon (15:30–16:30) to ensure a 4-h fasting period and to coincide with the completion of routine intravenous infusions. Participants removed metal objects and remained still during the measurement, with electrodes placed according to the manufacturer’s protocol (8). They were positioned supine with the upper limbs abducted 15° from the trunk and the legs spread to shoulder width, after at least 10 min of rest to allow for body fluid redistribution. Measurements were conducted in a temperature-controlled room.

#### 2.2.2 Diagnosis of sarcopenia

Sarcopenia was diagnosed based on the criteria established by the AWGS 2025. Initial screening was based on calf circumference (CC) and handgrip strength (HGS). Patients identified as high-risk proceed to the next phase of the study. For adults between 60 and 64 years of age, high-risk status was defined as CC < 34 cm and/or HGS < 34 kg for males, and CC < 33 cm and/or HGS < 20 kg for females. Reduced muscle mass was defined as an ASMI < 7.6 kg/m^2^ for males or <5.7 kg/m^2^ for females. For adults ≥65 years of age, high-risk status was defined as CC < 34 cm and/or HGS < 28 kg for males, and CC < 33 cm and/or HGS < 18 kg for females. Reduced muscle mass was defined as an ASMI < 7.0 kg/m^2^ and < 5.7 kg/m^2^, respectively. To ensure data accuracy, a second measurement was performed if the initial reading was deemed technically unsatisfactory.

#### 2.2.3 Diagnosis of malnutrition

Nutritional risk was screened using the Mini Nutritional Assessment - Short Form (MNA-SF). Individuals with an MNA-SF score ≤11 were considered at risk of malnutrition and subsequently underwent diagnosis of malnutrition based on the GLIM criteria. Diagnosis of malnutrition required the presence of at least one phenotypic criterion and one etiologic criterion. The phenotypic criteria included: (1) non-volitional weight loss (>5% within 6 months or >10% beyond 6 months); (2) low BMI (<18.5 kg/m^2^ for those aged <70 years, or <20.0 kg/m^2^ for those aged ≥70 years); and (3) reduced muscle mass. The etiologic criteria included: (1) reduced food intake or assimilation (<50% of energy requirements for >1 week, or any reduction for >2 weeks); and (2) inflammation or disease burden. Inflammation was identified by elevated levels of C-reaction protein (CRP), procalcitonin (PCT), or white blood cells count (14).

#### 2.2.4 Physical performance

SPPB score, which comprise assessment of gait speed, standing balance, and five times chair stand test. A total of score ≤ 9 was classified as impaired physical performance.

#### 2.2.5 Blood and biochemical data

Laboratory parameters were measured using fasting venous blood samples collected upon admission, including complete blood count, CRP, serum albumin, blood urea nitrogen (BUN), creatinine, uric acid (UA), and N-terminal pro-B-type natriuretic peptide (NT-pro BNP).

### 2.3 Statistic analysis

Continuous data are presented as mean ± standard deviation (SD) or median (interquartile range, IQR), and categorical data as frequencies (percentages). Group differences were analyzed using one-way ANOVA or the Kruskal–Wallis *H* test for continuous variables, and the chi-square or Fisher’s exact test for categorical variables.

The association between PhA and clinical outcomes (malnutrition and impaired physical performance) was evaluated using binary logistic regression models. Variables such as age, gender, BMI, and albumin, alongside those with *p* < 0.05 in the univariate analysis, were included in the multivariate models. Results are reported as adjusted odds ratios (aOR) with 95% confidence intervals (CI). To evaluate whether sarcopenia status modified the predictive value of PhA, an interaction term (PhA × sarcopenia) was incorporated into the models. Given the exploratory nature of the interaction analysis, a post-hoc power analysis was conducted to verify the adequacy of the sample size. Based on the observed effect size for the interaction term, the study achieved a statistical power of exceeding 80% at a two-sided significance level of 0.05, indicating that the sample size was sufficient to detect the reported interaction effects. The diagnostic accuracy of PhA for identifying malnutrition and impaired physical performance was evaluated using receiver operating characteristic (ROC) curve analysis, with the optimal cut-off was determined by the maximum Youden index. All analyses were performed using IBM SPSS Statistics (Version 23.0). A two-tailed *p* < 0.05 was considered statistically significant.

## 3 Results

### 3.1 Characteristics of participants

A total of 235 patients (median age 71) were enrolled in this cross-sectional study, of whom 96 (40.9%) were female. According to the AWGS 2025 criteria, the prevalence of sarcopenia was 48.5% (*n* = 114). Patients in the sarcopenia group exhibited significantly lower PhA values compared to those in the non-sarcopenia group (4.01 ± 0.85° vs. 4.91 ± 0.75°, *p <* 0.001). Across the five defined categories, the distribution of disease types showed no statistically significant differences between the non-sarcopenia and sarcopenia groups (*p* = 0.103). Furthermore, marked differences were observed between the two groups in terms of anthropometric parameters (e.g., BMI and ASMI), and several laboratory examinations (e.g., albumin and NLR). Detailed participant characteristics and inter-group comparisons are summarized in Table 1.

**Table 1:** Patient demographic and clinical characteristics stratified by sarcopenia.

	All (*n* = 235)	Non-sarcopenia (*n* = 121)	Sarcopenia (*n* = 114)	*p*
Age, years	71 (8)	69 (7.5)	73 (9.25)	<0.001
Female, *n* (%)	96 (40.90)	53 (43.80)	43 (38.05)	0.586
PhA (°)	4.51 ± 0.90	4.91 ± 0.75	4.01 ± 0.85	<0.001
BMI, kg/m^2^	22.87 ± 3.67	24.59 ± 3.57	21.29 ± 3.49	<0.001
Diseases category				0.103
AECOPD, *n* (%)	59	24 (19.83)	35 (30.70)	
ILD, *n* (%)	63	31 (25.62)	32 (28.07)	
LSOL, *n* (%)	49	28 (23.14)	21 (18.42)	
Pneu, *n* (%)	29	19 (15.70)	10 (8.77)	
Others, *n* (%)	35	19 (15.70)	16 (14.04)	
CC, cm	32.19 ± 3.41	34.01 ± 3.16	30.34 ± 2.71	<0.001
Male	32.64 ± 3.35	34.49 ± 3.33	30.91 ± 2.56	<0.001
Female	31.55 ± 3.40	33.37 ± 2.82	29.46 ± 2.71	<0.001
HGS, kg	22.15 ± 8.33	26.33 ± 7.82	17.69 ± 6.34	<0.001
Male	26.15 ± 7.72	31.26 ± 6.22	21.04 ± 5.41	<0.001
Female	16.34 ± 5.22	19.77 ± 3.93	12.55 ± 3.62	<0.001
ASMI, kg/m^2^	5.89 (0.98)	6.63 (0.99)	5.42 (0.73)	<0.001
Male	6.38 (0.86)	7.01 (0.75)	5.83 (0.56)	<0.001
Female	5.29 (0.65)	5.53 (0.66)	4.79 (0.46)	<0.001
SARC-calf positive (score ≥11), *n* (%)	86 (36.60)	17 (14.05)	71 (62.28)	0.002
MNA-SF,score, *n* (%)				<0.001
12–14	121 (51.49)	82 (67.77)	39 (34.21)	
≤11	114 (48.51)	39 (32.23)	75 (65.79)	
Hemoglobin, g/L	129 (25)	128.5 (10.25)	126 (18)	0.523
Male	135 (21)	133.9 (16.37)	129.8 (19.03)	0.73
Female	123 (19)	120.3 (17.16)	121.47 (17.58)	0.748
Albumin, g/L	37.58 ± 5.70	38.79 ± 4.59	36.73 ± 5.53	0.002
NLR	2.58 (2.35)	2.23 (1.92)	2.99 (3.0)	< 0.001
CRP, mg/L	3.37 (5.13)	2.81 (8.31)	3.79 (14.59)	0.032

PhA, phase angle; BMI, body mass index; AECOPD, acute exacerbation of chronic obstructive pulmonary disease; ILD, interstitial lung disease; LSOL, lung space-occupying lesions; Pneu, pneumonia as the primary diagnosis; Others: bronchiectasis, bronchial asthma, and pulmonary embolism; CC, calf circumference; HGS, hand grip strength; ASMI, appendicular skeletal muscle mass index; SARC-calf, SARC-F combined with calf circumference; MNA-SF, Mini Nutritional Assessment - Short Form; NLR, neutrophil-to-lymphocyte ratio; CRP, C-reactive protein.

### 3.2 Relationship between PhA, malnutrition and physical performance

Among the cohort, 102 (43.4%) patients were diagnosed with malnutrition based on the GLIM criteria, and 96 (40.9%) exhibited impaired physical performance (SPPB score ≤9). Table 2 shows the distribution of these outcomes. Notably, patients with sarcopenia demonstrated a significantly higher burden of both malnutrition and impaired physical performance compared to non-sarcopenic individuals (*p* < 0.001).

**Table 2:** Prevalence of malnutrition and impaired physical performance stratified by sarcopenia status.

	Total (*n* = 235)	Non-sarcopenia (*n* = 121, 51.1%)	Sarcopenia (*n* = 114, 48.5%)	*p*-value
The distribution of malnutrition				<0.001
NN *n* (%)	133 (56.6)	102 (84.3)	31 (27.2)	
MN *n* (%)	102 (43.4)	19 (15.7)	83 (72.8)	
The distribution of physical performance				<0.001
NPP *n* (%)	139 (59.1)	94 (77.7)	45 (39.5)	
IPP *n* (%)	96 (40.9)	27 (22.3)	69 (60.5)	

NN, normal nutrition; MN, malnutrition; NPP, normal physical performance; IPP, impaired physical performance.

**Figure 2:**
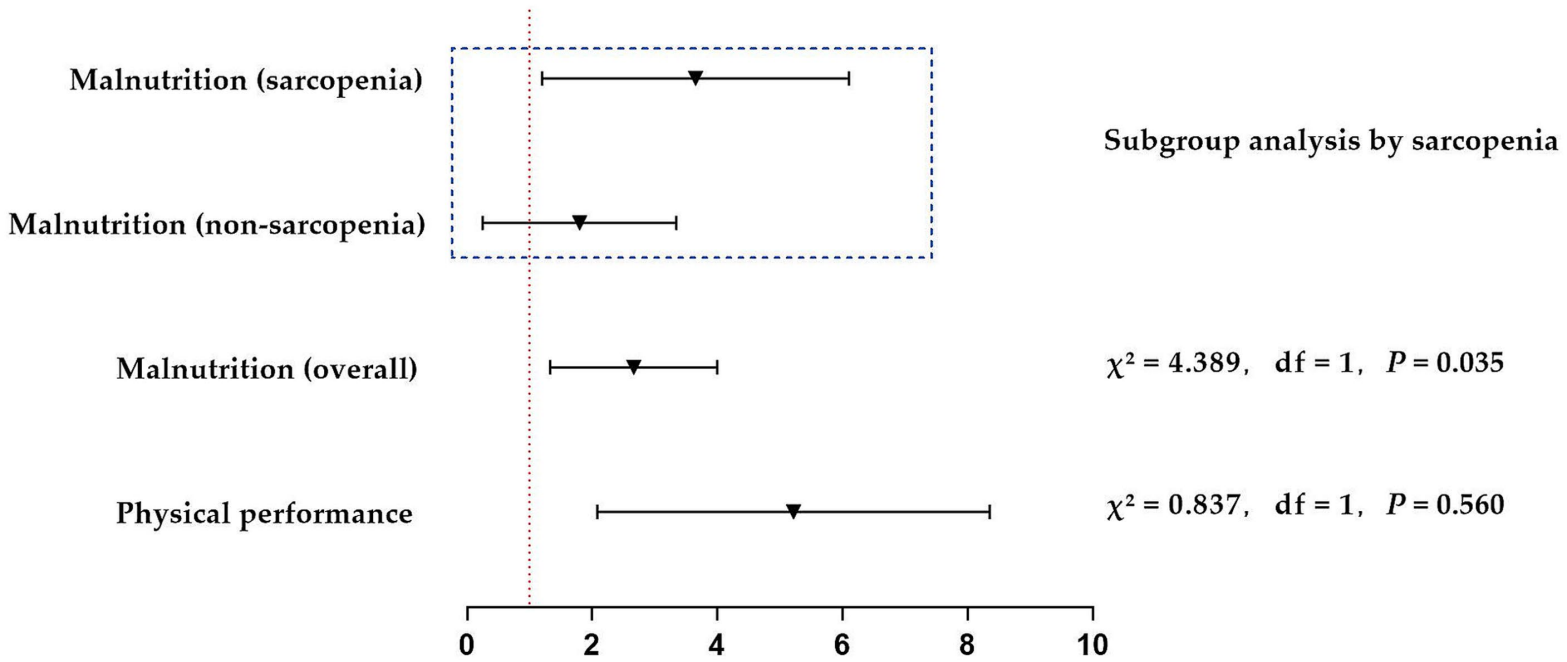
Interaction effects between PhA and sarcopenia on malnutritionrisk and physical performance. The blue box indicates subgroup analysis stratified by sarcopenia status. χ^2^ indicates the Wald chi-square statistic; df indicates degrees of freedom.

To investigate the potential moderating effect of sarcopenia for malnutrition and physical performance, we performed a formal interaction analysis (PhA × sarcopenia). The results revealed a significant interaction between PhA and sarcopenia status regarding malnutrition (*P interaction* = 0.035). In contrast, PhA demonstrated a consistent and robust association with impaired physical performance, independent of sarcopenia status (*P interaction =* 0.560). These findings suggest that PhA serves as a universal marker for physical performance, whereas its value for nutritional assessment is contingent upon muscle status. Details summarizes in Figure 2.

Multivariate logistic regression analysis further clarified these relationships (Table 3). Regarding malnutrition, subgroup analyses further demonstrate that low PhA was significantly associated with an increased risk of malnutrition in the sarcopenia group (aOR = 3.11, 95% CI: 1.52–6.33), after adjusting for age, sex, BMI, and albumin. However, this association was not statistically significant in the non-sarcopenia subgroup (aOR = 1.35, 95% CI: 0.52–3.52). These findings suggest that the association between PhA and malnutrition was primarily driven by the sarcopenia subgroup. In contrast, for impaired physical performance, lower PhA was independently associated with a higher risk of functional impairment across the entire cohort after adjusting for age, sex, BMI, albumin, and sarcopenia status (aOR = 4.59, 95% CI: 2.44–8.62).

**Table 3:** Multivariate logistic regression analysis of PhA as an independent predictor of malnutrition and physical performance.

Clinical outcomes	Population group	Adjusted OR (95% CI)	*p*-value
Malnutrition	Total population	2.44 (1.46–4.10)	0.002
	Sarcopenia	3.11 (1.52–6.33)	<0.001
	Non-sarcopenia	1.35 (0.52–3.52)	0.524
Impaired physical performance	Total population	4.59 (2.44–8.62)	<0.001

**Figure 3:**
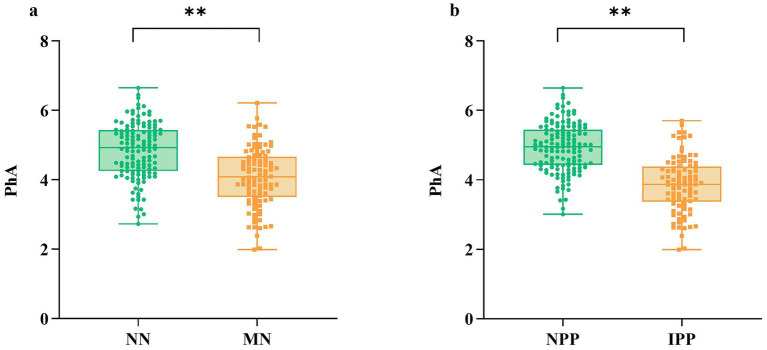
Comparison of PhA values across different nutritional statuses **(A)** and physical performance categories **(B)**. **: *p* < 0.001. NN, normal nutrition; MN, malnutrition; NPP, normal physical performance; IPP, impaired physical performance.

Regarding nutritional status, PhA was significantly lower in the malnutrition group compared to the normal nutrition group (4.07 ± 0.86° vs. 4.84 ± 0.79°, *p* < 0.001). Similarly, patients with impaired physical performance exhibited lower PhA values than those with normal performance (3.85 ± 0.78° vs. 4.93 ± 0.69°, *p* < 0.001, Figure 3).

**Figure 4:**
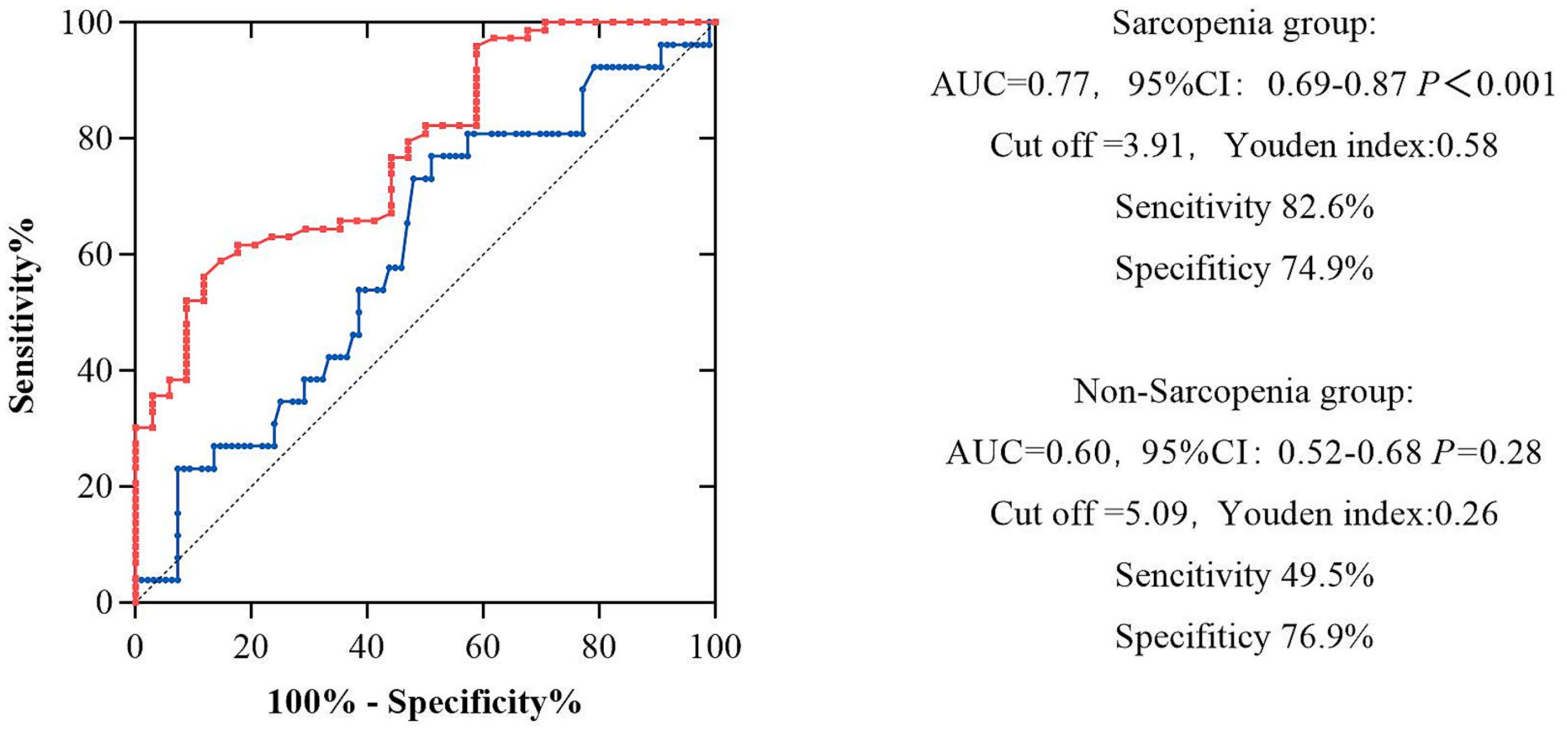
Receiver operating characteristic curves of PhA for identifying malnutrition, stratified by sarcopenia status.

The identification value of PhA for malnutrition, stratified by sarcopenia status, is illustrated in Figure 4. For the sarcopenia group, PhA demonstrated a moderated discriminative power with an AUC of 0.77 (95% CI: 0.69–0.87, optimal cutoff: 3.91°, sensitivity: 82.6%, specificity: 74.9%). In contrast, its diagnostic utility was limited in the non-sarcopenia group, with an AUC of 0.60 (95% CI: 0.52–0.68, optimal cutoff: 5.09°, sensitivity: 49.65%, specificity: 76.9%).

**Figure 5:**
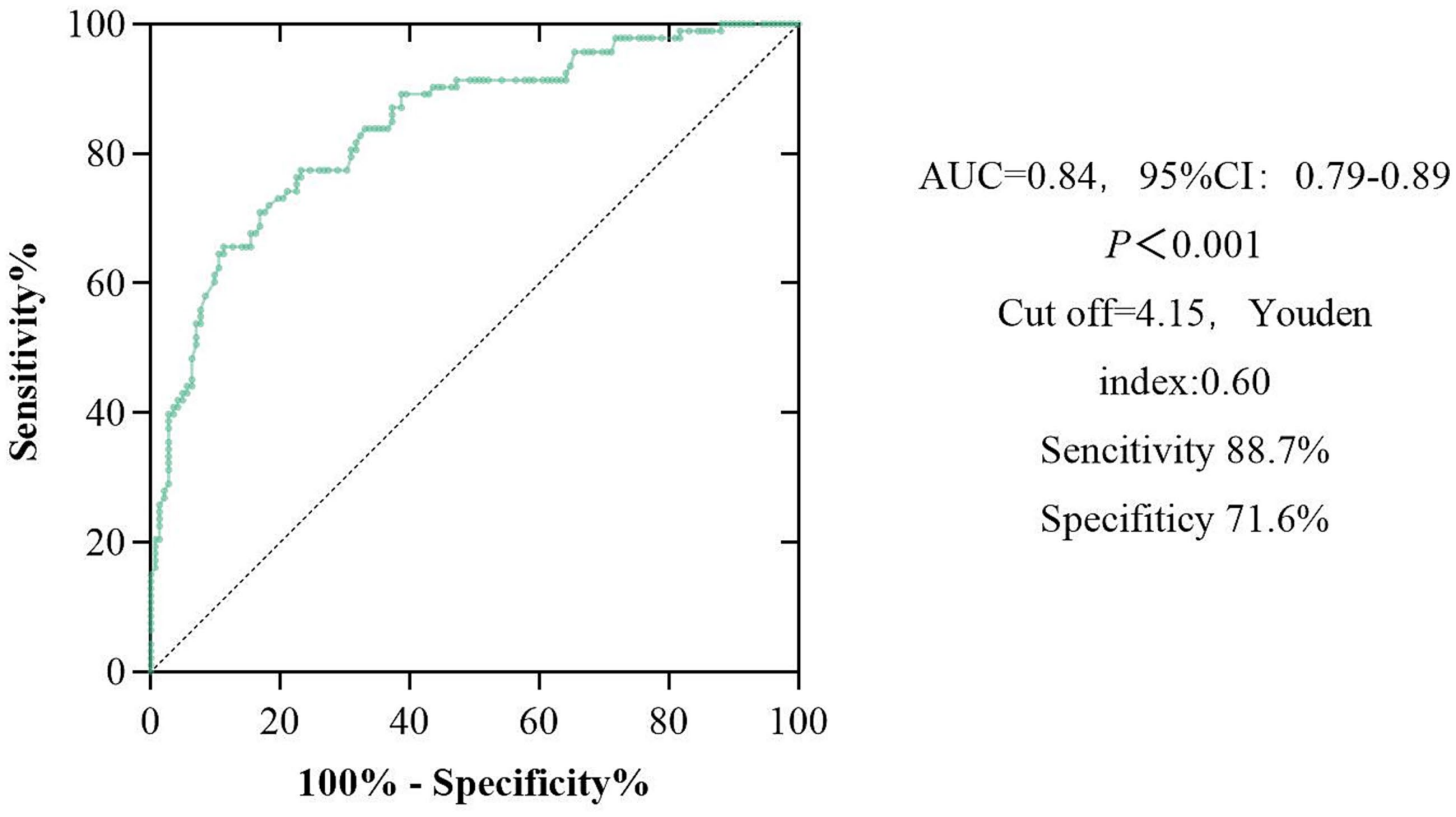
Receiver operating characteristic curves of PhA for identifying impaired physical performance in the total cohort.

For the assessment of physical performance across the entire cohort (Figure 5), PhA demonstrated a robust diagnostic efficacy with an AUC of 0.84 (95% CI: 0.79–0.89). The cutoff value for identifying impaired physical performances was 4.15° (sensitivity: 88.7%, specificity: 71.6%).

### 3.3 The relationship between PhA and other parameters

**Figure 6:**
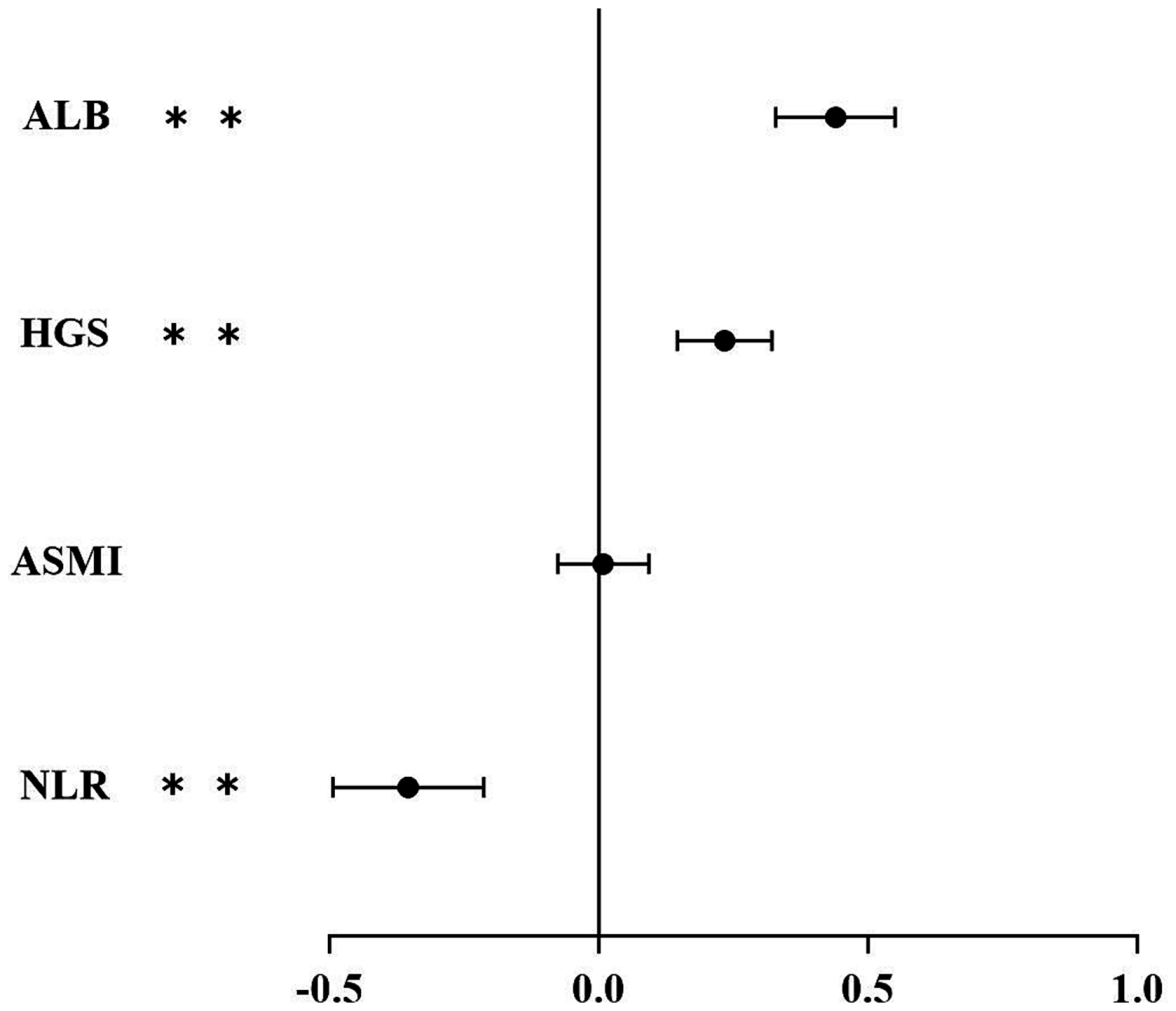
Forest plot of regression coefficients for the association between the phase angle and selected parameters. **: *p* < 0.001.

To further explore the clinical correlates of PhA, multivariable linear regression analyses were conducted. After adjusting for potential confounders, PhA demonstrated significant positive associations with serum albumin (*β* = 0.439, 95% CI: 0.328–0.550, *p* < 0.001), HGS (β = 0.233, 95% CI: 0.145–0.321, *p* < 0.001), and ASMI (*β* = 0.008, 95% CI: −0.076 to 0.092, *p* = 0.849). Conversely, a significant negative correlation was observed between PhA and the NLR (*β* = −0.362, 95% CI: −0.514 to −0.210, *p* < 0.001). These linear relationships are visually summarized in Figure 6.

## 4 Discussion

To the best of our knowledge, this is the first study to suggest that the identifying utility of PhA for malnutrition is significantly moderated by sarcopenia status among old adults with respiratory diseases. Our primary finding indicates that while PhA shows a robust and independent association with impaired physical performance regardless of muscle mass status, its utility in identifying malnutrition (via GLIM criteria) appears to be significantly modulated by the presence of sarcopenia. Specifically, the association between lower PhA and increased risk of malnutrition was notably stronger and only statistically significant in patients with sarcopenia. These results imply that PhA is a stable marker for physical performance, but its application in nutritional screening may depends on the current state of the muscle-cell reservoir.

The prevalence of sarcopenia based on AWGS 2025 was 48.5% among the 235 older patients. This figure is consistent with previous reports, which have estimated the prevalence of sarcopenia in similar clinical settings to range from 31.6 to 50% (15, 16). By adopting the AWGS 2025 criteria, our study ensures that the identified prevalence is reflective of the most current clinical understanding of muscle health in the Asian population. Sarcopenia is associated with diverse outcomes, including limitations in activities of daily living, cognitive decline, increased risk of falls, fractures, and premature death (17). In respiratory patients, there were several causes for sarcopenia. First, nutritional status has been identified as a key driver of sarcopenia. Strong associations between respiratory function and sarcopenia-related phenotypes are anticipated, especially physical performance and muscle mass (18). Second, the hypoxia pattern lead to increase respiratory muscle load and dyspnea that decrease the activity resulting in muscle deconditioning and ultimately, sarcopenia (19). Furthermore, systemic inflammation, common in chronic respiratory diseases, may further accelerate protein catabolism in skeletal muscle.

Consistent with the high prevalence in patients with respiratory diseases, we found a malnutrition rate of 43.4%, which consistent with previous report 41.1% in ILD patients (20). The diagnostic process of our study involved initial utilized via the MNA-SF, followed by the application of the GLIM criteria for definitive diagnosis. Protein-calorie malnutrition increases the risk of adverse clinical outcomes, such as diminished lung function (FEV1 predicted), impaired exercise capacity, respiratory failure, mechanical ventilation, sepsis, prolonged hospital stays, and increased mortality (20–22). In this study, impaired physical performance according to SPPB criteria was 40.9%. Reduced physical mobility is common in patients with respiratory diseases and is closely linked to prognosis and readmission. Meta analysis reported that the patients with COPD exhibit significantly slower gait speed than healthy controls, potentially elevating their risk of falls and a higher likelihood of hospital readmission (23). Patients with respiratory diseases frequently present with comorbidities including cardiovascular diseases, type 2 diabetes mellitus and sarcopenia. These studies suggested that exercise capacity is not only related to ventilatory function, but also to overall cardiovascular adaptation, glucose homeostasis (24, 25). Slower four-meter gait speed at hospital discharge, a physical performance measure, has been shown to be an independent predictor of higher 12-month readmission and mortality risk, establishing it as a valuable tool for risk stratification in AECOPD patients (26).

PhA serves as a robust biological indicator of cellular integrity and carries inherent prognostic significance. Due to its non-invasive, cost-effective, and straightforward nature, PhA is highly adaptable for different clinical environments. As an objective metric with high sensitivity and specificity, it facilitates the establishment of prognostic cutoffs and the longitudinal monitoring of therapeutic efficacy (12). Previous studies have reported that PhA is correlated with body composition, physical function complications, length of ICU stay, survival rate, symptoms, and quality of life (27, 28). PhA is also an significant indicator in heart failure, obesity, liver diseases, respiratory diseases, chronic kidney diseases and so on (29–33). However, the diagnostic utility of PhA for nutritional assessment remains a subject of debate. While some researchers found only a weak correlation between PhA and nutritional status (27), while others have demonstrated that PhA exhibits fair to good diagnostic accuracy when using the GLIM criteria (34–37). The prior reports linking low PhA with malnutrition may not have accounted for the muscle-mass dependency. In our study, we used an interaction term between PhA and sarcopenia status in multivariate models and found that although PhA independently predicts impaired physical performance regardless of muscle mass status, its utility in identifying malnutrition (based on GLIM criteria) is significantly moderated by the presence of sarcopenia. Our findings provide a novel perspective to resolve this clinical discrepancy by identifying sarcopenia status as a pivotal moderator.

A notable finding of this study is the divergent utility of PhA in identifying malnutrition and impaired physical performance. Its association with malnutrition differs by sarcopenia status, while its link with physical performance remains robust and consistent across the entire cohort. Although PhA was significantly associated with malnutrition in the overall population, our subgroup analysis suggests that this relationship was more pronounced in the sarcopenia subgroup. This discrepancy likely reflects the distinct pathophysiological drivers behind these two outcomes. The differential value of PhA for malnutrition imply its context different distribution, which is heavily influenced by the patient’s underlying disease status. As demonstrated by Di Vincenzo et al. (38), PhA does not simply increase linearly with body mass; rather, it often reaches a plateau in individuals with higher weight or preserved muscle mass. Furthermore, Plauth et al. (39), hypothesized that the existence of a minimum biological threshold for PhA, below which physiological viability is severly compromised. Our findings align with this concept. The mean PhA in the sarcopenia group (4.01°) clusters tightly around the malnutrition cutoff (3.91°), suggesting these patients have reached a critical “low PhA plateau”. Since sarcopenic patients are already nearing this critical threshold of cellular compromise, PhA may serve as a highly sensitive indicator of nutritional status. In this vulnerable subgroup, where patients are physiologically closer to this lower limit, even minor nutritional deficits might be accompanied by detectable cellular membrane changes, thus amplifying the observed association between PhA and malnutrition. Therefore, PhA becomes a highly sensitive marker for the “tipping point” of malnutrition, resulting in a significant OR: 3.11 (1.52–6.33). Conversely, patients without sarcopenia have not yet reached this depletion threshold. Their greater lean tissue mass provides a metabolic reserve that uncouples PhA values from acute nutritional failure, explaining the lack of statistical significance and the lower AUC (0.60) in this subgroup. While the narrow gap in our cohort may reflect the specific characteristics of our study population, it underscores the sensitivity of PhA in identifying high-risk individuals. This necessitates a stratified approach when using PhA to assess nutritional risk in respiratory patients. Interestingly, the utility of PhA for physical performance is independent of sarcopenia status. We hypothesized that in this population, physical performance may be more heavily influenced by systemic or extramuscular factors, such as cardiopulmonary function and systemic oxygen delivery, rather than being determined primarily by skeletal muscle status.

There are several limitations in this study. First, this was a single-center study with a limited sample size. Our cohort included patients with five different respiratory diseases from the same clinical department, rather than a single disease. Owing to the modest sample size, we did not perform further subgroup analyses stratified by specific diseases. Larger cohorts would enable such analyse in the future. Although our post-hoc power analysis indicated sufficient statistical power, future large-scale prospective studies with pre-specified sample size estimations are warranted to validate our findings. Second, the divergence in the association between PhA and malnutrition based on sarcopenia status may be specific to this cohort and might not be directly applicable to other pathologies or different ethnic groups. Third, due to the lack of long term follow-up, we were unable to investigate the relationship between PhA, nutritional transitions, and changes in physical performance. Moreover, because of the cross-sectional design of this study, causal relationships between variables cannot be inferred, and the observed associations should be further examined in longitudinal studies. In future, multi-center, large-scale trials involving divers diseases are necessary. Long term follow-up studies are essential to investigated the dynamic change between PhA, malnutrition and impaired physical performance.

## 5 Conclusion

The effectiveness of phase angle (PhA) as a nutritional assessment tool in respiratory patients is contingent upon skeletal muscle status; specifically its utility in nutritional assessment appears more pronounced in the presence of sarcopenia. In contrast, PhA shows a consistent and robust association with physical performance across the entire population, regardless of muscle status. Consequently, we propose that PhA may be a valuable metric for evaluating physical performance in respiratory departments. However, its application for malnutrition screening should be interpreted with caution and ideally stratified by the patient’s musculoskeletal health.

## Data Availability

The original contributions presented in the study are included in the article/supplementary material, further inquiries can be directed to the corresponding author/s.
